# The role of B cell-activating factor system in autoimmune diseases: mechanisms, disease implications, and therapeutic advances

**DOI:** 10.3389/fimmu.2025.1538555

**Published:** 2025-06-06

**Authors:** Liang Li, Shengxian Shen, Shuai Shao, Erle Dang, Gang Wang, Hui Fang, Hongjiang Qiao

**Affiliations:** Department of Dermatology, Xijing Hospital, Fourth Military Medical University, Xi’an, Shaanxi, China

**Keywords:** B cell-activating factor, autoimmune diseases, targeted therapies, autoimmunity, b cells, T cells

## Abstract

The B cell-activating factor (BAFF) system, comprising two ligands and three receptors, plays a pivotal role in adaptive and innate immunity, driving autoimmunity through dysregulated B and T cell survival, differentiation, and cytokine production. This review synthesizes evidence linking BAFF system overexpression to multiple autoimmune diseases, including systemic lupus erythematosus (SLE), Sjögren’s syndrome (SS), bullous pemphigoid (BP), pemphigus vulgaris (PV), and alopecia areata (AA), where elevated BAFF system molecule levels correlate with autoantibody titers, disease activity, and post-B cell depletion relapse. BAFF-targeted therapies have demonstrated efficacy in reducing disease activity in SLE and SS. Key challenges include interspecies receptor expression discrepancies and context-dependent signalling cascades. Emerging strategies, such as sequential therapy with rituximab followed by belimumab, show promise in treating refractory autoimmune diseases such as BP and PV by counteracting the post-depletion BAFF surge. Despite progress, mechanistic gaps in BAFF-mediated crosstalk between innate and adaptive immunity, as well as interspecies-specific pathogenesis warrant further investigation using humanized disease models and single-cell transcriptomic profiling. This review underscores the therapeutic potential of BAFF system modulation while advocating for disease-specific clinical trials to optimize precision-therapeutic targeting in autoimmune diseases.

## 1 Introduction

The B cell-activating factor (BAFF) system can be synthesized in various cell types and consists of two ligands, BAFF and A proliferation-inducing ligand (APRIL), along with three receptors, BAFF receptor (BAFFR), B cell maturation antigen (BCMA), and transmembrane activator and calcium modulator and cyclophilin ligand interactor (TACI) (1). As a key component of the BAFF system, BAFF was originally discovered as a fundamental survival cytokine for B cells. BAFF can maintain B cell survival, autoreactive B cell selection, class switch recombination, and the maintenance of long-lived plasma cells (PC) (2). Additionally, increasing evidence has shown that the BAFF system can promote T cell survival, differentiation, as well as regulate other immune cells (3–8).

Research in both humans and mouse models has identified that the BAFF system is a vital player in autoimmunity pathogenesis (9, 10). Overexpression of BAFF system molecules has been detected in patients with various types of autoimmune diseases, including systemic lupus erythematosus (SLE), Sjögren’s syndrome (SS), systemic sclerosis (SSc), bullous pemphigoid, pemphigus vulgaris, and alopecia areata, and may be involved in the pathogenesis of these diseases (11–20). Therefore, BAFF system molecules are considered potential therapeutic targets for autoimmune diseases. Monoclonal antibodies have been developed to target single or dual BAFF system molecules, and belimumab has been approved for use in SLE (21). Moreover, several clinical studies on these mAbs are underway, with positive results for SS and SSc (22–24).

In this review, we describe the key structural and biological features of the BAFF system as well as its functional implications in the pathogenesis of autoimmune diseases. We also highlight that therapies targeting the BAFF system are a promising strategy for treating different autoimmune diseases and warrant further investigation.

## 2 B cell-activating factor system

### 2.1 Ligands

B cell-activating factor (BAFF) and its structural homologue A proliferation-inducing ligand (APRIL) are type II transmembrane proteins belonging to the tumour necrosis factor (TNF) cytokine superfamily (25, 26). Although they share the same structure, their functions are quite different. BAFF can be synthesized as a membrane-bound form (mBAFF) and converted into a secreted form (sBAFF), which is the main form in circulation (27, 28). BAFF is mainly expressed by immune cells, such as monocytes, macrophages, dendritic cells, and neutrophils (29, 30). Moreover, BAFF can be produced by non-immune cells, including adipocytes, keratinocytes, and intestinal epithelial cells (31, 32). BAFF expression is upregulated by interleukin (IL)-10, interferons (IFNs), toll-like receptor (TLR) agonists, granulocyte colony-stimulating factor (G-CSF), and by the activation of interferon regulatory factors (IRFs), such as IRF1 and IRF2. Conversely, IRF4 and IRF8 negatively regulate BAFF expression (33, 34). APRIL can also be synthesized by various types of immune cells in both membrane-bound and secreted form. Similarly, APRIL expression is regulated by IL-10, IFNs, G-CSF, TLR, and IRFs (9, 35–37).

### 2.2 Receptors

BAFF receptor (BAFFR), B cell maturation antigen (BCMA), and transmembrane activator and calcium modulator and cyclophilin ligand interactor (TACI) are type III membrane proteins with distinct but complementary effects (38, 39). These three receptors, like their ligands, can be converted from the membrane-bound form into the secreted form (40–42). BCMA and TACI share two common ligands, BAFF and APRIL, whereas BAFFR has only one ligand, BAFF (43, 44). Moreover, BCMA shows a lower affinity for BAFF compared with that for APRIL, whereas TACI has an equal affinity for both ligands (45). In contrast to the widespread expression of ligands, the expression of these three receptors is restricted to specific immune cells. Although B cells can express all three receptors, their expression varies during B cell maturation stage (37, 46, 47). In contrast to B cells, human T cells only express BAFFR, whereas mouse T cells express BAFFR and TACI (47–49).

### 2.3 B cell-activating factor function in autoimmunity

**Figure 1:**
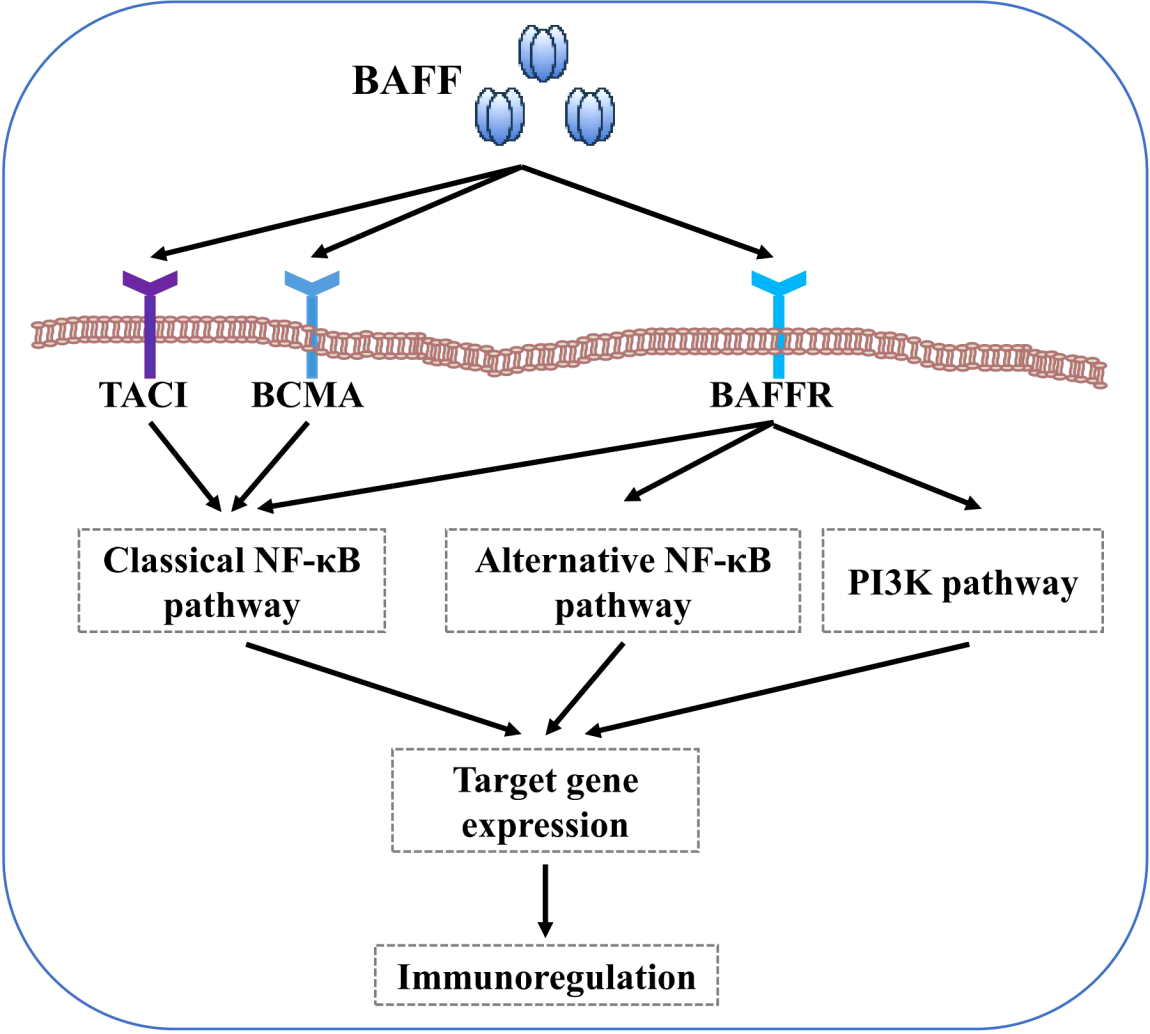
BAFF signaling pathways. BAFF binding to BAFFR activates both the classical and alternative NF-κB pathways. In contrast, BAFF interaction with BCMA and TACI only activates the classical NF-κB pathway. Furthermore, BAFF binding to BAFFR also activates the PI3K pathways.

B cell-activating factor (BAFF) system molecules modulate a variety of biological processes, including cell survival, differentiation, and other effector functions, as demonstrated in some autoimmune diseases, such as systemic lupus erythematosus (SLE), Sjögren’s syndrome (SS), and systemic sclerosis (SSc) (50–52). BAFF receptor (BAFFR) interaction with BAFF activates both the classical and alternative transcription factor nuclear factor-kappa B (NF-κB) pathways, whereas B cell maturation antigen (BCMA) and transmembrane activator and calcium modulator and cyclophilin ligand interactor (TACI) linked to BAFF only activates the classical NF-κB pathway (29, 53). Furthermore, BAFF activates the phosphoinositide-3-kinase (PI3K) dependent signalling cascade to support cell survival (54) (**Figure 1**). However, most immunobiological findings related to molecules of the BAFF system have been obtained using transgenic mouse models.

#### 2.3.1 Function of BAFF in adaptive immunity

**Figure 2:**
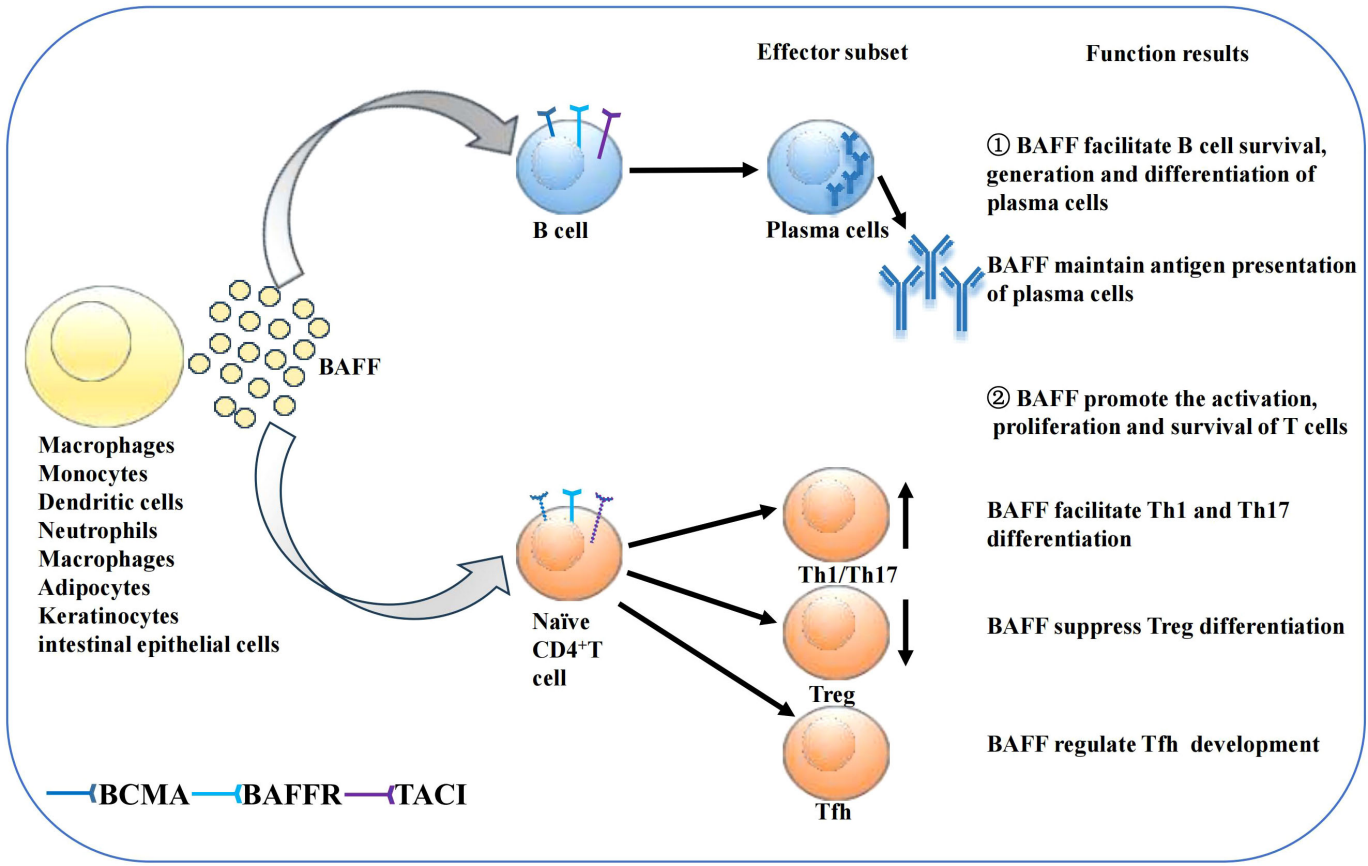
Function of BAFF on B cells and T cells. Secreted BAFF can be synthesized by several kinds of cells as shown in the figure. BAFF then promotes B cell survival, generation and differentiation of plasma cells, supports antigen presentation of PCs. BAFF also affects T cell survival and proliferation. BAFF may promote the differentiation of Th1 and Th17 cells while suppressing Treg differentiation. Moreover, BAFF regulate Tfh development.

BAFF system molecules play an important role in adaptive immunity (**Figure 2**). As discussed in Section 2.2, the differential expression of the three receptors during B cell maturation stage is related to their individual functions. BAFFR expression begins in transitional B cells, preventing premature apoptosis via BAFFR-dependent pro-survival signals (47, 55–58). BCMA expression is restricted to long-lived bone marrow plasma cell (PC) and plasmablasts, where it supports PC formation, maintenance, and differentiation while preserving antigen presentation (59–63). TACI is mostly expressed by marginal zone B cells, activated B cells, switched memory B cells, and PCs. TACI negatively regulates early B cell maturation and mediates PC generation, maintenance, and differentiation, as well as T cell-independent immunoglobulin (Ig) isotype conversion and release (64–68). Mouse models underscore BAFF’s essential role in B cell physiology, responsiveness, and autoimmunity. BAFF transgenic mice (BAFF Tg mice), which overexpress BAFF, exhibit SLE and SS-like manifestations, such as increased peripheral mature B cell numbers, immune globulin (Ig) deposits in the kidney, and enlarged lymphoid organs (69). Conversely, BAFFR-mutant or BAFF-deficient mice show significantly reduced peripheral mature B cells and impaired immune responses (38, 70–72). Compared to BAFF, APRIL overexpression or APRIL deficiency does not cause remarkable abnormalities during B cell maturation (73, 74). Additionally, APRIL-deficient mice show impaired class-switching to IgA and enhanced IgG responses to T-dependent antigens (33, 75). TACI-deficient mice exhibit pseudo-autoimmune traits, with increased B cell numbers, elevated autoantibody-producing cells, and diminished T cell-independent humoral responses, indicating that TACI may negatively regulate B cells (65, 76, 77). Finally, the vital role of BCMA in the survival of long-term bone marrow PCs has been confirmed in BCMA-deficient mice (59). Despite significant advances in understanding BAFF’s effects on B cells, many aspects require further investigation.

The BAFF system also affects T cell activation, proliferation, and survival by acting as a costimulatory signal together with TCR in both effector and naïve T cells (4, 78, 79). BAFF-deficient mice develop reduced quantities of effector T cells, while APRIL-deficient mice show normal proliferation, differentiation, and T cell function (74, 80). BAFF augments T cell stimulation by increasing costimulatory molecules expression in antigen-presenting cells and by upregulating the expression of BAFFR and TACI in murine T cells (3, 81). BAFF, which interacts with BAFFR on T cells, can promote the activation and proliferation of CD4^+^ T cells through the PI3K/Akt pathway (3, 49). However, treatment with anti-BAFFR antibodies enhances the cytolytic function of human CD4^+^ and CD8^+^ T cells, a discrepancy likely attributable to varying BAFF concentrations (82). Notably, BAFF may facilitate T-helper (Th) 1 and Th 17 cell differentiation and suppress regulatory T cell differentiation (83–86). Moreover, the BAFF system regulates follicular helper T (Tfh) cells through the noncanonical NF-κB pathway by mediating the expression of inducible costimulatory ligand expression on B cells (87). TACI-deficient mice exhibit increased quantities of Tfh cells in their spleens after T cell-dependent antigen immunisation, largely owing to the upregulation of inducible costimulatory ligand on TACI-deficient B cells (88). However, the effect of the BAFF system on T cells remains unclear and requires further investigation.

#### 2.3.2 Function of BAFF in innate immune cells

Although evidence is minimal, BAFF also affects other immune cells. Monocytes, which are a source of BAFF, can also be regulated by BAFF. Along with augmented release of proinflammatory cytokines, BAFF strongly promotes monocyte survival and differentiation into macrophages by activating the NF-κB pathway (6, 89). It induces human myeloid dendritic cell maturation, increasing costimulatory molecule expression and inflammatory cytokine secretion (7). Furthermore, regulation of the BAFF system in megakaryocytic cells and natural killer (NK) cells has also been reported. However, no BAFF receptors are expressed on NK cells, suggesting that NK cells are indirectly regulated by BAFF (8, 90, 91).

#### 2.3.3 Future directions on BAFF

Although BAFF system molecules play a central role in regulating adaptive immunity, their context-specific mechanisms remain poorly understood. A major translational challenge arises from ​species-specific differences in receptor expression: BAFFR is found exclusively on human T cells, whereas murine models show co-expression of BAFFR and TACI in T cells. These discrepancies complicate the extrapolation of findings from animal studies to human biology. Additionally, paradoxical observations—such as anti-BAFFR antibodies enhancing human T cell cytotoxicity while BAFF simultaneously promotes Th17 polarization—highlight the complexity of BAFF-mediated signaling pathways. To bridge this translational gap, B-hBAFF/hBAFFR transgenic mouse models​ could provide a critical platform. These models would allow researchers to simulate human-specific receptor signaling by selectively blocking interference from TACI in murine T cells. Such systems would be particularly valuable for studying BAFF-driven pathologies like SLE -associated kidney damage or SS salivary gland dysfunction, enabling direct validation of therapeutic targets in a humanized context. Cutting-edge technologies like ​spatial transcriptomics combined with single-cell ATAC-seq​ could further clarify the spatiotemporal dynamics of BAFFR, TACI, and BCMA expression within disease microenvironments. For example, mapping these receptors in SLE renal follicular regions might reveal how they coordinate with Tfh cell expansion and plasma cell differentiation, offering mechanistic insights into disease progression. Beyond adaptive immunity, BAFF also influences innate immunity by modulating myeloid cells and indirectly regulating NK cells. However, its dual roles in driving inflammation versus supporting tissue repair remain unclear. Future studies should prioritize ​myeloid-specific BAFFR knockout models​ to dissect BAFF’s effects on macrophage and dendritic cell function. Equally important is investigating ​receptor-independent BAFF-NK interactions, such as potential signaling through heparan sulfate proteoglycans or extracellular vesicles, which could uncover novel immunoregulatory pathways. By addressing these gaps, researchers can unravel BAFF’s multifaceted roles in immunity and inflammation, paving the way for targeted therapies in autoimmune and inflammatory diseases.

## 3 Pathogenic role of the B cell-activating factor system in autoimmune diseases

**Figure 3:**
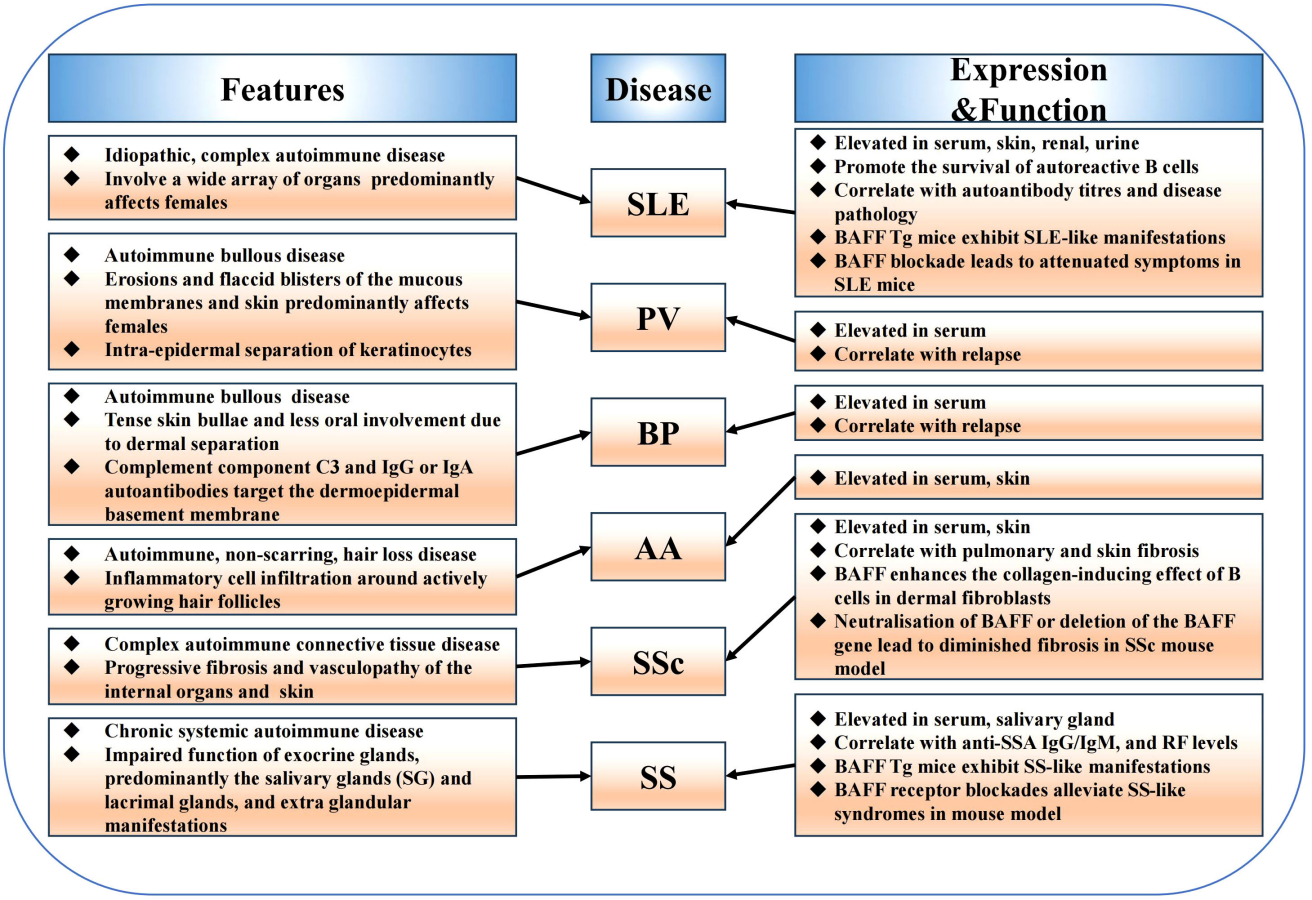
The function of BAFF system in autoimmune diseases. BAFF system molecules are elevated in various autoimmune diseases including systemic lupus erythematosus, Sjögren’s syndrome, systemic sclerosis, bullous pemphigoid, pemphigus vulgaris, alopecia areata, and are involved in the pathogenesis of these diseases. Furthermore, mouse models have highlighted the role of the BAFF system in regulating immune cells, promoting systemic autoimmunity, and mediating the occurrence and development of autoimmune diseases.

Overexpression of B cell-activating factor (BAFF) system molecules has been detected in patients with various types of autoimmune diseases, such as systemic lupus erythematosus (SLE), Sjögren’s syndrome (SS), systemic sclerosis (SSc), bullous pemphigoid (BP), pemphigus vulgaris (PV), and alopecia areata (AA) (**Figure 3**). Additionally, elevated circulating BAFF levels correlate with autoantibody titers in patients with SLE and SSc. Furthermore, studies on animal models have highlighted that BAFF system molecules participate in regulating immune cells, promoting systemic autoimmunity, and mediating the occurrence and development of autoimmune diseases. Here, we discuss the expression and the roles of BAFF system molecules in autoimmune diseases pathogenesis (**Table 1**).

**Table 1:** Expression of BAFF system molecules in autoimmune diseases.

Disease	location	BAFF	APRIL	BAFFR	BCMA	TACI	Reference
SLE	Serum	Elevated	Elevated	/	Elevated	Elevated	(12, 40, 41, 92)
	Skin	Elevated	no mention	Elevated	Elevated	/	(13, 93, 94)
	Renal	Elevated(mRNA)	Elevated	/	Elevated(mRNA)	Elevated(mRNA)	(95)
	Urine	Elevated	Elevated	no mention	no mention	no mention	(96)
SS	Serum	Elevated	Elevated	no mention	no mention	no mention	(18, 19)
	Salivary gland	Elevated	/	no mention	no mention	/	(18, 19, 97, 98)
SSc	Serum	Elevated	Elevated	Elevated	no mention	Elevated	(16, 17, 99, 100)
	Skin	Elevated(mRNA)	no mention	no mention	no mention	no mention	(16)
BP	Serum	Elevated	Elevated	no mention	no mention	no mention	(11, 15)
PV	Serum	Elevated	/	no mention	no mention	no mention	(14, 15, 101)
AA	Serum	Elevated	/	no mention	no mention	no mention	(102–104)
	Skin	Elevated	no mention	no mention	no mention	no mention	(104)

AA, alopecia areata; APRIL, A proliferation-inducing ligand; BAFF, B cell-activating factor; BAFFR, BAFF receptor; BCMA, B cell maturation antigen; BP, bullous pemphigoid; PV, pemphigus vulgaris; SLE, systemic lupus erythematosus; SS, Sjögren’s syndrome; SSc, systemic sclerosis; TACI, transmembrane activator and calcium modulator and cyclophilin ligand interactor.

### 3.1 Systemic lupus erythematosus

Systemic lupus erythematosus (SLE) is an idiopathic, complex autoimmune disease that involves a wide array of organs and predominantly affects females (105). Approximately 70% of the patients experience some degree of skin involvement (106). Elevated BAFF levels have been detected in the skin, serum, urine, and kidneys of patients with SLE, with correlations to disease pathology and autoantibody titres in both human and murine models (12, 107–110). However, the correlation between circulating BAFF levels and anti-double-stranded DNA (anti-dsDNA) titers remains controversial. This contradiction may be attributed to the different detection methods (111, 112). BAFF Tg mice with BAFF overexpression exhibit SLE-like manifestations, such as hypergammaglobulinemia, increased serum immune complexes, levels of rheumatoid factor (RF) and anti-dsDNA, as well as renal Ig deposits (69). Moreover, BAFF overexpression has been observed in other spontaneous SLE-prone mice (39). Consistently, BAFF blockade attenuates symptoms and disease activity in SLE mice, thereby improving survival (113, 114). Excess BAFF may participate in the development of SLE through supporting autoreactive B cells survival (115). Lupus nephritis (LN) is a severe complication of SLE, characterized by kidney inflammation due to autoimmune-mediated damage, primarily affecting the glomeruli. Glomerular APRIL and BAFF levels are significantly elevated in patients with LN (95). Furthermore, BAFF levels in the kidneys of LN mice are correlated with disease activity and the histopathological activity index (116). BAFF promotes LN by inducing a tertiary lymphoid structure in the kidney and modulating the position of glomerular T cells (117). In addition to LN, cutaneous symptoms are also observed in many patients with SLE. The levels of BAFF and its three receptors—BAFFR, BCMA, and TACI—are increased in patients with cutaneous lupus erythematosus (CLE). In these patients, BAFF is mainly expressed in keratinocytes, whereas the three receptors are mainly expressed in the lymphoid cells. Moreover, BAFF expression is significantly upregulated after stimulation with immunostimulatory DNA motifs in cultured keratinocytes (13, 94, 118).

### 3.2 Sjögren’s syndrome

Sjögren’s syndrome (SS) is a chronic systemic autoimmune disease characterized by impaired function of exocrine glands and extra glandular manifestations (119). Notably, patients with SS exhibit elevated serum and SG levels of BAFF. In addition, increased BAFF levels are strongly correlated with anti-SSA IgG/IgM and RF levels (18, 98, 120). As mentioned in Section 3.1, BAFF Tg mice overexpressing BAFF exhibit SLE-like manifestations. However, as they age, these mice acquire features of SS, such as glandular inflammation and structural destruction (18). Early BAFFR blockade alleviates SS-like syndromes in mice (121). Moreover, salivary epithelial cells in patients can secrete different forms of BAFF to participate in the pathogenesis of SS, and BAFF can also promote epithelial cell survival through autocrine signalling (122). Additionally, studies in patients with SS and mouse models have shown that BAFF is involved in the formation of germinal centre-like structures, which are important in SS pathogenesis (123–126).

### 3.3 Systemic sclerosis

Systemic sclerosis (SSc) is a complex autoimmune connective tissue disease characterized by progressive fibrosis and vasculopathy of the internal organs and skin (127). The serum levels of APRIL and BAFF are increased in patients with SSc and positively correlated with skin and pulmonary fibrosis, respectively (16, 17). Furthermore, adding anti-IgM and BAFF to a co-culture of dermal fibroblasts and peripheral B cells isolated from patients with SSc showed that BAFF enhanced the collagen-inducing effect of B cells in dermal fibroblasts (128). Tight skin (TSK/+) mice, which are genetic a murine model of SSc, develop cutaneous fibrosis and autoimmunity. Intriguingly, circulating BAFF levels and skin fibrotic cytokines are elevated in the TSK/+ mouse model. Moreover, BAFF antagonists enhance the expression of anti-fibrotic cytokines, therefore inhibiting autoantibody production, skin fibrosis, and fibrotic cytokine expression in TSK/+ mice (129). Notably, neutralisation of BAFF or deletion of the BAFF gene led to diminished fibrosis in a bleomycin-induced model of pulmonary fibrosis (130). Furthermore, a small pilot study using BLM to suppress BAFF showed an improvement in skin hardening in patients with SSc (51). The results of these studies show that the pathogenesis of SSc is complicated and involves various environmental and genetic factors, warranting further investigation in future studies.

### 3.4 Bullous pemphigoid

Bullous pemphigoid (BP) is an autoimmune bullous skin disease characterized by tense skin bullae and less oral involvement. These manifestations may be due to the presence of complement component C3 and IgG autoantibodies, which target the dermo–epidermal basement membrane, resulting in dermal separation (131). One study revealed increased levels of circulating BAFF in patients with BP (15). Furthermore, flow cytometric analysis confirmed elevated BAFF expression in memory and naïve B cells in patients with BP (132). Conversely, another study found that the levels of circulating BAFF molecules in healthy controls were comparable with those in patients with BP (133). This discrepancy is likely attributable to differences in disease duration. Serum APRIL levels are also increased in patients with BP (11). RTX is a third-line treatment for BP and has clinical benefits for severe BP. One study found that serum BAFF level increased after RTX treatment in patients with BP. Additionally, serum BAFF levels increased before the peripheral B cell number returned to normal, implying a relapse (134). Dipeptidyl peptidase 4 inhibitors (DPP4is), commonly used for the treatment of type 2 diabetes, increase the risk of BP (DPP4is-associated BP). BAFF expression is higher in regular BP skin (not associated with DPP4is) compared to that in DPP4i-associated BP skin (135). However, few studies have explored the role of BAFF in BP pathogenesis, with studies largely restricted to phenomenological observations. Thus, further investigation is required to determine whether and how BAFF is involved in BP pathogenesis.

### 3.5 Pemphigus vulgaris

Pemphigus vulgaris (PV) is an autoimmune bullous disease characterized by erosions and flaccid blisters of the skin and the mucous membranes owing to the intra-epidermal separation of keratinocytes (136). While some studies report elevated serum APRIL levels in patients with PV, others show no significant differences, casting doubt on its role (11, 14). A similar inconsistency is observed with serum BAFF levels in PV (15, 101, 137), likely due to variations in sample sizes across studies. Rituximab (RTX), a targeting B-lymphocyte CD20 mAb, is the most common B cell-depleting therapy for bullous dermatoses. One study found that circulating BAFF levels were significantly increased after RTX treatment, which normalised upon the recovery of peripheral CD19^+^ B cells (138). Another reported higher baseline BAFF levels in patients with PV than in healthy controls, with levels rising further after 3 months of RTX treatment, suggesting a link between BAFF and PV immunopathogenesis (137). Although the B cell-depleting agent RTX is effective for patients with PV, relapses are frequent. Hebert et al. observed that most relapses occurred precisely when autoreactive B cells reappeared and BAFF serum levels increased, suggesting that relapse after RTX therapy might be attribute to a raise in circulating BAFF levels and the reappearance of autoreactive B cells (101). Taken together, the role of the BAFF system in PV remains unclear, and further studies are required to elucidate its role in PV.

### 3.6 Alopecia areata

Alopecia areata (AA) is a non-scarring autoimmune hair loss disorder characterized by inflammatory cell infiltration around actively growing hair follicles (20). Circulating BAFF levels are elevated in patients with AA with more than three lesions (102). This phenomenon was confirmed by a later study, which also revealed increased tissue BAFF levels in patients with AA. Furthermore, BAFF and TH17 synergistically participate in the pathogenesis of AA (104). While research on BAFF’s role in AA remains limited, its significance cannot be overlooked.

## 4 Targeting B cell-activating factor system molecules for autoimmune diseases therapy

**Figure 4:**
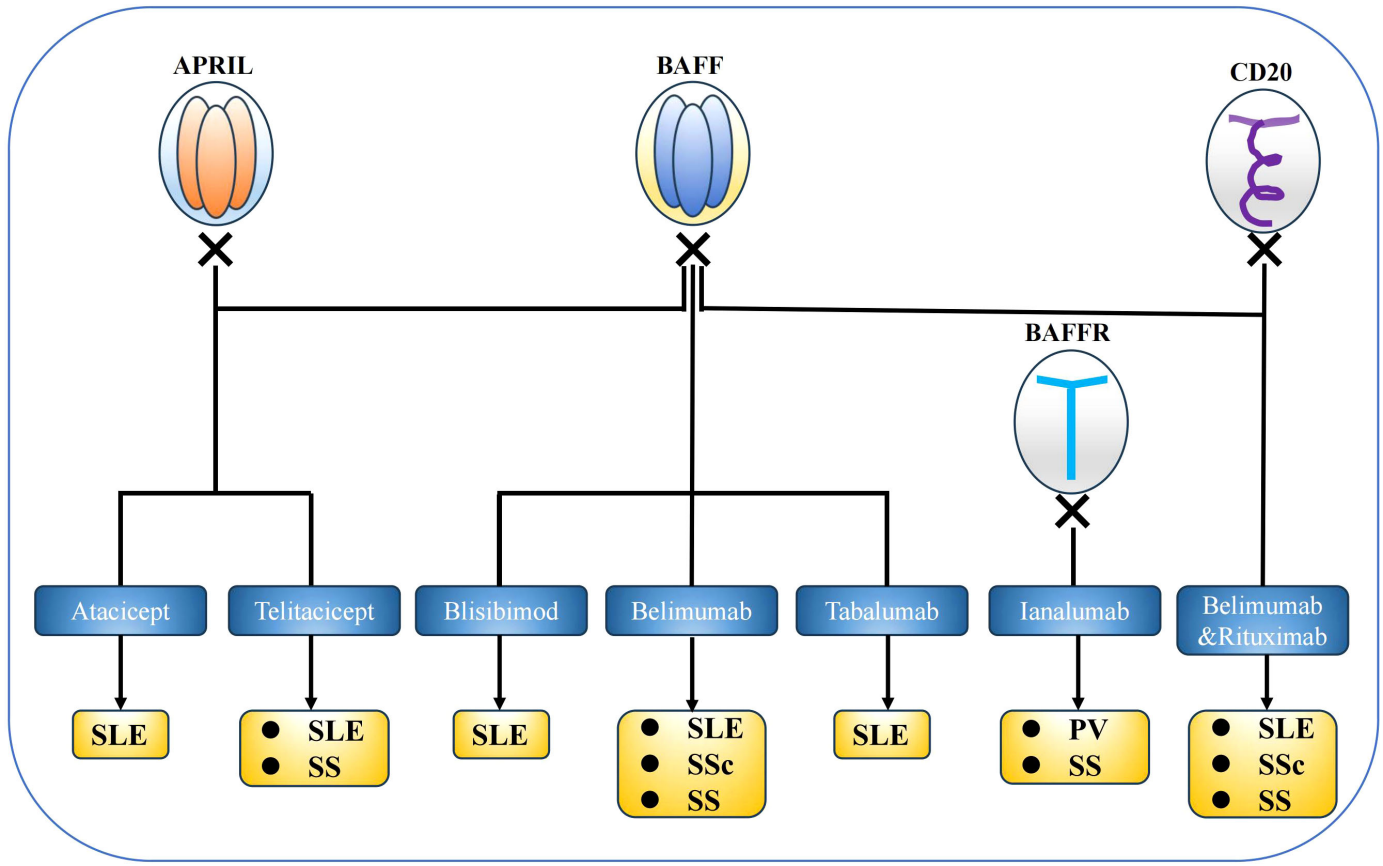
BAFF system targeted therapies. Several experiments that target BAFF system molecules have been conducted in mouse models and clinical settings, and belimumab was approved for systemic lupus erythematosus therapy. BAFF system molecules are considered potential targets for the treatment of several autoimmune diseases.

Since their discovery in 1999, studies targeting single or dual B cell-activating factor (BAFF) system molecules have been conducted (**Figure 4**). Belimumab (BLM) is a fully humanised recombinant IgG1λ mAb that antagonises the biological activity of sBAFF by preventing its interaction with receptors. BLM was approved for systemic lupus erythematosus (SLE) therapy in 2011, suggesting that BAFF system molecules are potential targets for the treatment of several autoimmune diseases. Several clinical trials are ongoing, with encouraging results for sjögren’s syndrome (SS) and systemic sclerosis (SSc), reinforcing BAFF-targeting therapies as a potential strategy for autoimmune diseases treatment. Below, we summarize several BAFF system-targeting therapies for autoimmune diseases (**Table 2**).

**Table 2:** BAFF targeted clinical trials in autoimmune diseases.

Agent	Target	Disease	Trail registration number	Number of participants	Status	Phase	Reference
Belimumab	BAFF	SLE	NCT00657007	70	Completed	Phase 1	(139)
			NCT00071487	449	Completed	Phase 2	(140)
			NCT00410384.	819	Completed	Phase 3	(141)
			NCT00424276	865	Completed	Phase 3	(142)
			NCT01484496 NCT00724867	839 268	Completed Completed	Phase 3 Phase 3	(143, 144) (145)
			NCT01639339	448	Completed	Phase 3	(146)
		SS	NCT01160666	20	Completed	Phase 2	(23, 147)
			NCT01008982	15	Completed	Phase 2	(23, 147)
		SSc	NCT01670565	20	Completed	Phase 2	(22)
Ianalumab (VAY736)	BAFFR	SS	NCT02149420	27	Completed	Phase 2	(148)
			NCT02962895	192	Completed	Phase 2	(149)
			NCT05350072	285	Recruiting	Phase 3	(150)
			NCT05349214	489	Recruiting	Phase 3	(151)
		PV	NCT01930175	13	Terminated (strategic reasons)	Phase 2	(152)
Atacicept	BAFF and APRIL	SLE	NCT00624338	461	Completed	Phase 2	(153, 154)
			NCT01972568	306	Completed	Phase 2	(155, 156)
			NCT02070978	253	Terminated (shortage of drug supply)	Phase 2	(157, 158)
			NCT05609812	360	Recruiting	Phase 3	(159)
Telitacicept (RC18)	BAFF and APRIL	SLE	NCT02885610	249	Completed	Phase 2	(160, 161)
			NCT04082416	335	Completed	Phase 3	(162)
			NCT05306574	341	Recruiting	Phase 3	(163)
		SS	NCT04078386	42	Completed	Phase 2	(164)
			NCT05673993	37	Not yet recruiting	Phase 3	(165)
Belimumab & Rituximab	BAFF&CD20	SLE	NCT02260934	43	Completed	Phase 2	(166)
			NCT03312907	292	Completed	Phase 3	(167, 168)
			NCT03747159	70	Recruiting	Phase 3	(169)
		SS	NCT02631538	86	Completed	Phase 2	(170)
		SSc	NCT03844061	30	Recruiting	Phase 2	(171)

APRIL, A proliferation-inducing ligand; BAFF, B cell-activating factor; BAFFR, BAFF receptor; PV, pemphigus vulgaris; SLE, systemic lupus erythematosus; SS, Sjögren’s syndrome; SSc, systemic sclerosis.

### 4.1 B cell-activating factor-targeted therapies

The efficacy and safety of belimumab (BLM) in systemic lupus erythematosus (SLE) were validated across multiple pivotal clinical trials (139–146). In phase III trials for active SLE, intravenous BLM combined with standard therapy significantly outperformed placebo in SLE Responder Index (SRI) rates at week 52 in the BLISS-76 and BLISS-SC trials (141, 142). Subcutaneous administration further improved outcomes in moderate-to-severe SLE, with an SRI-4 response rate of 61.4% vs 48.4% and a 49% reduction in severe flare risk (143). Notably, hypocomplementemic/anti-dsDNA^+^ patients exhibited enhanced benefits alongside corticosteroid-sparing effects (144). Long-term extension data confirmed sustained efficacy over 7 years: SRI response increased from 41.9% (year 1) to 75.6% (year 7), with 31.4% mean prednisone reduction and 83.2% CD20^+^ B cell depletion, while maintaining stable safety profiles (145). In LN management, BLM combined with standard therapy significantly improved primary and complete renal responses, reducing renal-related event/death risk by 49% (146). These findings led to FDA approval for SLE in 2011 and an expanded indication for active LN in 2020, cementing BLM as a key LN therapy (146, 172).

The use of BLM in patients with sjögren’s syndrome (SS) has also been assessed. In a clinical study, there was a significant improvement in clinical manifestations and biomarkers of B cell activation in patients with SS who were treated long-term with BLM (23, 147). Furthermore, a clinical study assessed the safety and efficacy of BLM in patients with SSc who received background mycophenolate mofetil. This study found a significantly improvement of clinical symptoms in the BLM group compared with the placebo group (22). Overall, the results are encouraging and justify further randomised controlled studies with larger populations.

The use of BLM after rituximab (RTX) for the treatment of SLE with bullous pemphigoid (BP) has also been reported. For example, one patient with SLE overlapping with BP achieved significant clinical remission and steroid sparing after RTX-BLM sequential treatment, suggesting that a combination therapy of anti-CD20 and anti-BAFF mAbs might maintain longer B cell depletion and clinical remission (172). In addition, a case of pemphigus vulgaris (PV) was successfully treated with BLM after a failed steroid therapy course. After four cycles of BLM treatment, clinical symptoms and autoantibody levels were significantly decreased in the patient, highlighting the effectiveness of BLM in treating PV (173).

### 4.2 B cell-activating factor receptor-targeted therapies

Ianalumab (VAY736), a fully human anti-B cell-activating factor receptor (BAFFR) mAb, has two action mechanisms: direct depletion of BAFFR^+^ B cells and competitive BAFFR blockade, leading to B cell apoptosis (149). In a clinical study, ianalumab was used as a single-dose treatment for patients with sjögren’s syndrome. The results showed that ianalumab reduced clinical manifestations, B cell activation biomarker expression, and serum Ig light chain levels and augmented the salivary flow rate in sjögren’s syndrome (148). Moreover, a dose-finding study confirmed a dose-related decrease in disease activity (149). However, Novartis terminated a clinical trial of VAY736 in patients with pemphigus vulgaris prior to its completion for strategic reasons (152, 174).

### 4.3 B cell-activating factor- and a proliferation-inducing ligand-targeted therapies

#### 4.3.1 Atacicept

Atacicept is a recombinant soluble fusion protein that targets both B cell-activating factor (BAFF) and a proliferation-inducing ligand (APRIL) (175). A phase II study showed that patients with systemic lupus erythematosus (SLE) administered with 150 mg atacicept experienced a lower flare rate than those administered with a placebo (153). Moreover, a *post-hoc* analysis of this study demonstrated a dose–response relationship between atacicept concentrations and reduced flare rates, which further confirmed the efficiency of atacicept (154). Additionally, a phase IIb ADDRESS II trial demonstrated the dose-dependent efficacy of atacicept (75/150 mg) in treating active SLE, with 75 mg achieving a significant SRI-4 response and reduced flare risk in high-activity subgroups, while maintaining placebo-comparable safety (155, 156). Subsequently, a long-term extension of this study was conducted. Although it was terminated early owing to drug supply shortage, atacicept exhibited an acceptable safety and efficacy during the study period (157, 158). Although these clinical trials yielded positive results, further research is needed to evaluate the safety and effectiveness of atacicept for treating SLE.

#### 4.3.2 Telitacicept

Telitacicept (RC18) is a novel, recombinant transmembrane activator and calcium modulator and cyclophilin ligand interactor-Fc fusion protein. Similar to atacicept, telitacicept can bind to BAFF and APRIL simultaneously. The safety and efficacy of telitacicept have been assessed in patients with SLE in a phase IIb trial, and it succeeded in meeting the primary endpoint, validating its safety (160, 161). Telitacicept was approved for treating active SLE in China in 2021 (176). A subsequent phase III randomised, double-blind, placebo-controlled trial involving 335 patients demonstrated robust efficacy and safety of weekly subcutaneous telitacicept combined with standard therapy compared to placebo (162). These promising results have driven global multicentre clinical trials, some of which are currently recruiting. Moreover, the use of telitacicept in patients with SS was assessed in a phase II study and yielded positive results, with significant improvements in clinical manifestations (164). Ongoing phase III trials aim to further evaluate its efficacy (177).

### 4.4 Belimumab and rituximab combination therapy

Despite the demonstrated efficacy of rituximab (RTX) in autoimmune diseases therapy, frequent relapses remain a clinical challenge. Several studies have investigated this issue, revealing that serum B cell-activating factor (BAFF) levels rise significantly during B cell depletion post-RTX and return to baseline upon B cell recovery. Given that excessive BAFF has been shown to rescue self-reactive B cells from apoptosis, these findings suggest that the recovery of self-reactive B cells may be attributed to elevated BAFF levels following B cell depletion (178, 179). These findings led to investigations of sequential therapy with RTX followed by belimumab (BLM). For instance, a multicentre randomised trial in patients with refractory lupus nephritis demonstrated that adding BLM to RTX/cyclophosphamide therapy was safe and modulated B cell reconstitution more effectively than B cell depletion alone (166). However, the phase III BLISS-BELIEVE trial found that sequential subcutaneous BLM with a single RTX cycle did not achieve superior disease control at week 52 or clinical remission at week 64 compared to BLM plus placebo. Nonetheless, this combination significantly reduced anti-dsDNA antibodies, modulated B cell subsets, and prolonged disease control duration, warranting further investigation (167, 168). In sjögren’s syndrome (SS), sequential RTX-BLM therapy improved clinical outcomes compared to monotherapies without compromising safety (170, 180). Collectively, although current studies on sequential therapy are limited, these findings highlight its potential as a promising therapeutic strategy that warrants further research to explore its long-term effects and mechanisms.

Emerging BAFF-targeted therapies for systemic lupus erythematosus (SLE), SS, and systemic sclerosis show translational promise in understudied autoimmune diseases. In bullous pemphigoid (BP), elevated BAFF molecule levels in memory B cells and post-RTX surges correlate with relapse, while DPP4 inhibitor-associated cases exhibit reduced BAFF molecule levels, implicating pathogenic heterogeneity (134, 135). Dual BAFF/APRIL inhibitors and SLE-validated B cell maturation antigen chimeric antigen receptor T cell immunotherapy therapies may counteract BP autoantibody pathology. For pemphigus vulgaris, post-RTX BAFF resurgence aligns with B cell recovery (101, 137, 138); sequential use of RTX-BLM or transmembrane activator and calcium modulator and cyclophilin ligand interactor agonists could limit autoreactivity. In alopecia areata, BAFF elevation is linked to Th17 inflammation (104), supporting the potential for telitacicept or JAK/BAFF dual inhibition. Disease-specific trials are critical to refine BAFF-axis modulation strategies across autoimmune diseases.

## 5 Conclusions

The B cell-activating factor (BAFF) system plays an indispensable role in autoimmunity. Increasing numbers of clinical trials targeting BAFF antagonism have yielded promising results, leading to the approval of BLM for active systemic lupus erythematosus. However, despite these advances, the understanding of BAFF’s role in autoimmune diseases pathogenesis remains in its early stages, leaving many aspects yet to be explored. In terms of targeted BAFF therapy for autoimmune diseases, there is a shortage of available drugs; therefore, additional clinical trials with larger sample sizes are required to identify new targeted drugs.

## Glossary

AA: alopecia areata
anti-dsDNA: anti–double-stranded DNA
APRIL: A proliferation-inducing ligand
BAFF: B cell-activating factor
BAFFR: BAFF receptor
BAFF Tg mice: BAFF transgenic mice
BCMA: B cell maturation antigen
BLM: belimumab
BP: bullous pemphigoid
CLE: cutaneous lupus erythematosus
DPP4is: dipeptidyl peptidase 4 inhibitors
ESSDAI: European League Against Rheumatism Sjögren’s Syndrome Disease Activity Index
ESSPRI: European League Against Rheumatism Sjögren’s Syndrome Patient Reported Index
G-CSF: granulocyte colony-stimulating factor
Ig: immunoglobulin
IFNs: interferons
IL: interleukin
LN: lupus nephritis
IRFs: interferon regulatory factors
mAbs: monoclonal antibodies
mBAFF: membrane-bound form BAFF
NF-κB: nuclear factor-kappa B
NK: natural killer
PC: plasma cell
PI3K: phosphoinositide-3-kinase
PV: pemphigus vulgaris
RF: rheumatoid factor
RTX: rituximab
sBAFF: soluble form BAFF
SG: salivary gland
SLE: systemic lupus erythematosus
SS: Sjögren’s syndrome
SSc: systemic sclerosis
TACI: transmembrane activator and calcium modulator and cyclophilin ligand interactor
Tfh: follicular helper T
Th: T-helper
TLR: toll-like receptor
TNF: tumour necrosis factor
TSK/+: tight skin
